# Transplacental transfer of total immunoglobulin G and antibodies to *Plasmodium falciparum* antigens between the 24th week of gestation and term

**DOI:** 10.1038/s41598-022-21908-8

**Published:** 2022-11-07

**Authors:** Alexander K. K. Kayatani, Rose G. F. Leke, Robert I. J. Leke, Josephine Fogako, Diane Wallace Taylor

**Affiliations:** 1 Department of Tropical Medicine, Medical Microbiology and Pharmacology, John A. Burns School of Medicine, University of Hawaii at Manoa, 651 Ilalo Street, Bioscience Building, Honolulu, HI 96813 USA; 2 Faculty of Medicine & Biomedical Sciences, The Biotechnology Center, University of Yaoundé 1, Messa, BP 3851, Yaoundé, Cameroon

**Keywords:** Immunology, Microbiology

## Abstract

Full-term newborns have antibody (Ab) repertoires and levels similar to their mothers to help protect them from environmental pathogens. Unfortunately, preterm babies, especially those born < 34 weeks, have reduced levels of protective antibodies. In Africa, antibodies to *Plasmodium falciparum* are important in protection from malaria. This study investigated the transfer of total IgG and antibodies to 9 *P. falciparum* antigens and tetanus toxoid between 24 weeks and term. Paired maternal and cord samples from 166 preterm (24–37 weeks) and 154 term deliveries were used. Transfer efficiency was expressed as the ratio of Ab levels in cord to maternal plasma (CMR). At 24–25 weeks, CMR ranged from 0.31 to 0.94 for the different antigens; the rate of transfer was similar for all antigens between 24 and 40 weeks; resulting in median CMR of 0.49–0.95 at term. Babies of mothers with hypergammaglobulinemia and normal IgG levels had similar amounts of IgG, supporting data that saturation of the neonatal Fc-receptor occurs at ~ 16 mg IgG/ml. Thus, babies born prior to 34–35 weeks in Africa are likely to have reduced Ab levels to some, but not all antigens. Since IgG transfer is Fc-mediated, why differences exist in CMR among the antigens warrants further investigation.

## Introduction

Throughout pregnancy, IgG is actively transported across the placenta from the mother to the fetus^1,2^. Accordingly, full-term babies usually have IgG levels similar to their mothers to protect them during the first few months of life from pathogens in their environments. To ensure newborns have adequate protective levels of IgG, pregnant women have been immunized against preventable infectious diseases, i.e., measles and tetanus toxoid (TTx)^3,4^. Transplacentally-acquired IgG is especially important in developing countries where infants are exposed to multiple pathogens and access to pediatric care may be limited. One common childhood disease is malaria, caused by *Plasmodium falciparum. *In utero*-*acquired IgG is thought to play an important role in protecting infants against developing severe disease*.* Unfortunately, babies born prematurely, i.e., prior to completing 37 weeks of gestation, are likely to have lower levels of maternal IgG than term newborns^5–8^, potentially making them more susceptible to some infectious diseases, including malaria.

Transport of IgG transport across the placenta is an active process mediated by the neonatal Fc receptor (FcRn)^9^. IgG transport begins as early as 6 weeks of gestation and increases slowly until the 28th week^10^. Thereafter, IgG levels increase rapidly in fetal cord blood reaching maternal levels around 33–36 weeks^2,11,12^, i.e., the cord to mother ratio (CMR) of IgG (mg/ml) is ~ 1.0. Since transfer is an active process, CMR greater than 1.0 are common in term deliveries. At term, CMR for total IgG and pathogen-specific antigens have been well studied; however, establishing the rates of Ab increase during the second and early third trimesters have been challenging. Clearly, babies born prematurely have lower IgG levels than term babies, but the actual amount of IgG present between 24- and 37-weeks merits further investigation, especially for very premature newborns.

Studying transfer of maternal IgG to specific pathogens throughout pregnancy has been challenging; not only because of difficulties in obtaining samples, but also because not all women have antibodies (Abs) to the pathogen or allergen under investigation. The latter problem is eliminated in areas where *Plasmodium falciparum* malaria is endemic, since essentially all women have been repeatedly infected with malaria prior to pregnancy. Accordingly, these women have Abs to multiple *P. falciparum* antigens and are thus a valuable resource for studying transplacental transfer of Abs.

Unlike in the USA and Europe where CMR are usually ≥ 1.0 at term, studies in Africa have reported that total cord blood IgG levels are often lower than those of the mother, with CMR ranging from 0.4 to 1.0^13–17^. These results have been interpreted as evidence of transplacental insufficiency, most likely due to maternal hypergammaglobulinemia (HIgG) and/or sequestration of malaria parasites in the placenta (i.e., placental malaria (PM). In addition, the transfer of IgG Abs to pathogen-specific antigens are reported to be reduced, e.g., CMR for TTx and measles are < 1.0 at term^18–20^. Interestingly, Okoko and colleagues (2001) reported that both HIgG and/or PM reduced the transfer of IgG to some pathogens including herpes simplex, respiratory syncytial, varicella-zoster, but not to diphtheria toxoid, *S. pneumonia*, *Hemophilis influenza* type b, and TTx^21^. Likewise, CMR of Abs to some, but not all malarial antigens, may be reduced (Reviewed in^17^). Since IgG transport is an FcRn-mediated process^9^, one would predict that IgG Abs to different antigens, especially those of the IgG1 isotype, would be transferred at equal rates. Thus, it is surprising that transfer of IgG to some antigens, but not to others, is reduced. Accordingly, it is important to understand the dynamics and timing of transplacental-IgG transfer in the African setting.

The current study used paired mother-cord plasma samples from premature (24–37 week of gestation) and term deliveries collected in Yaoundé, Cameroon to (i) follow the increase in total IgG and Abs to 9 *P. falciparum* antigens and TTx between 24 weeks of gestation and term, (ii) compare the rate of transfer among the antigens during this period, and (iii) evaluate the influence of HIgG and PM on efficacy of transfer. The overall goal was to provide information on how to better manage the health of premature African newborns.

## Results

### Characteristics of mothers and babies

Table 1 provides information on the 320 mother-baby pairs used. The 166 women with preterm deliveries (PTD) averaged 24.9 ± 0.45 years of age, 28.9% (48/166) were primigravidae, and 29.5% (49/166) had PM (Table 1). Since all available samples from women with PTD were included, these data reflect the characteristics of women with PTD in the general population. As expected, neonatal birth and placental weights increased with gestational age (r = 0.98, *p* < 0.001 and r = 0.97, *p* < 0.001, respectively). An average birth weight of 1207 ± 142 g was recorded at 24–25 weeks followed by an increase (slope) of 126 gm biweekly until term. Pairs from 154 full-term deliveries (FTD) were randomly selected to have an equal number of women with (50.1%) and without (49.1%) PM. Women with FTD were of a similar age (averaged 24.9 ± 0.45 years) as women with PTD, but had higher percentage of primigravidae because placental malaria-positive (PM+) women are usually of lower gravidity (27.1% vs 40.9%, *p* = 0.013).

**Table 1:** Characteristics of mother and baby pairs used in the study (n = 320).

Weeks of gestation	N =	Mean maternal age (yr)^a^	% Primigravidae^b^	% Malaria-positive	Mean birth weight (gm)^a^	Mean placenta weigh (gm)^a^
**Weeks of gestation**						
Pre-term deliveries (n = 166)						
24–25	7	25.3 ± 0.8	0	0	1207 ± 142	386 ± 34
26–27	13	25.4 ± 0.5	15	15	1442 ± 171	370 ± 44
28–29	11	26.2 ± 0.6	27	27	1255 ± 116	384 ± 37
30–31	30	25.4 ± 0.3	30	33	1942 ± 143	459 ± 17
32–33	27	27.9 ± 0.2	26	37	2024 ± 108	488 ± 22
34–35	34	24.4 ± 0.1	29	26	2311 ± 95	515 ± 29
36–37	44	24.2 ± 0.1	34	32	2714 ± 66	551 ± 22
Full term deliveries (n = 154)						
38–39	62	24.6 ± 0.1	37	55^c^	3146 ± 72	595 ± 21
≥ 40	92	25.0 ± 0.1	43	48^c^	3302 ± 89	604 ± 13

^a^Means ± SEM are provided for maternal age, baby and placental birthweights.

^b^The difference in percent primigravidae between PTD (n = 166) and term deliveries (n = 154) was determined using Chi Square (*p* = 0.013).

^c^Samples from women with term deliveries (n = 154) were selected to include an equal number of women who were placental malaria-positive (PM+: 50.1%) and -negative (PM−: 49.1%).

### Timing of transplacental transfer of maternal IgG

**Figure 1:**
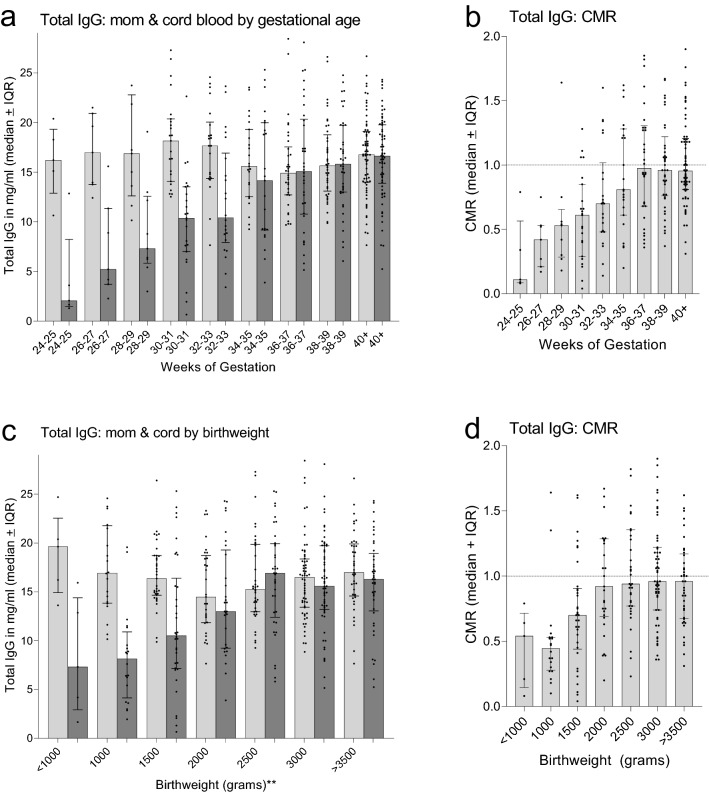
Timing of transfer of maternal IgG to the fetus based on data from premature and term deliveries. (**a**) Amount (mg/ml) of IgG in maternal and cord blood at two-week intervals. (**b**) Cord to maternal ratio (CMR) of IgG (median ± IQR). (**c**) Comparison of total maternal IgG in with different birthweights (**based on 500 g-intervals). (**d**) CMR of babies with different birthweights (based on 500-g intervals). Results are expressed as median ± IQR. Horizontal dotted lines indicate when maternal and cord IgG levels became equal (CMR = 1.0). n = 230 maternal and cord plasma pairs.

Total IgG levels were determined for the first 230 paired maternal-cord plasma samples that included 123 PTD and 107 FTD (Fig. 1). Throughout pregnancy, mothers had 16.2 mg IgG/ml blood (median ± IQR: 16.2 (13.8, 19.1). Although the amount appeared to be slightly lower late in pregnancy, no difference in IgG levels was found among the 9 gestational groups (Kruskal–Wallis (K–W) test *p* = 0.43) (Fig. 1a). In comparison, cord IgG levels increased linearly from a median of 2.1 mg/ml at 24–25 weeks to 16.6 mg/ml at 40 weeks, with an estimated rate of increase of 0.66 ± 0.08 mg/ml every two weeks. Median IgG levels in cord blood became similar to those of the mother between 34 and 37 weeks (Fig. 1a,b). When the results were expressed as CMR, CMR increased linearly from a median of 0.11 at 24–25 weeks to 0.98 at 36–37 weeks and remained the same through ≥ 40 weeks (Fig. 1b). Since maternal IgG levels remained stable during pregnancy, CMR directly reflected the increase of IgG levels in cord blood.

Because it was difficult to accurately assign gestational age, especially when ultrasound was not available, IgG levels in cord blood were evaluated in newborns with different birthweights (Table 1). As expected, median cord IgG concentrations (mg/ml) and CMR increased with birthweight (MFI: r = 0.94, *p* = 0.0015; CMR: r = 0.89, *p* = 0.007, respectively). At approximately 2000–2500 g birthweight (Fig. 1c), cord blood IgG levels were similar to the mothers (Fig. 1d).

### Transplacental transfer of maternal IgG Abs to 9 malarial antigens and TTx

#### Timing of antigen-specific IgG transfer

**Figure 2:**
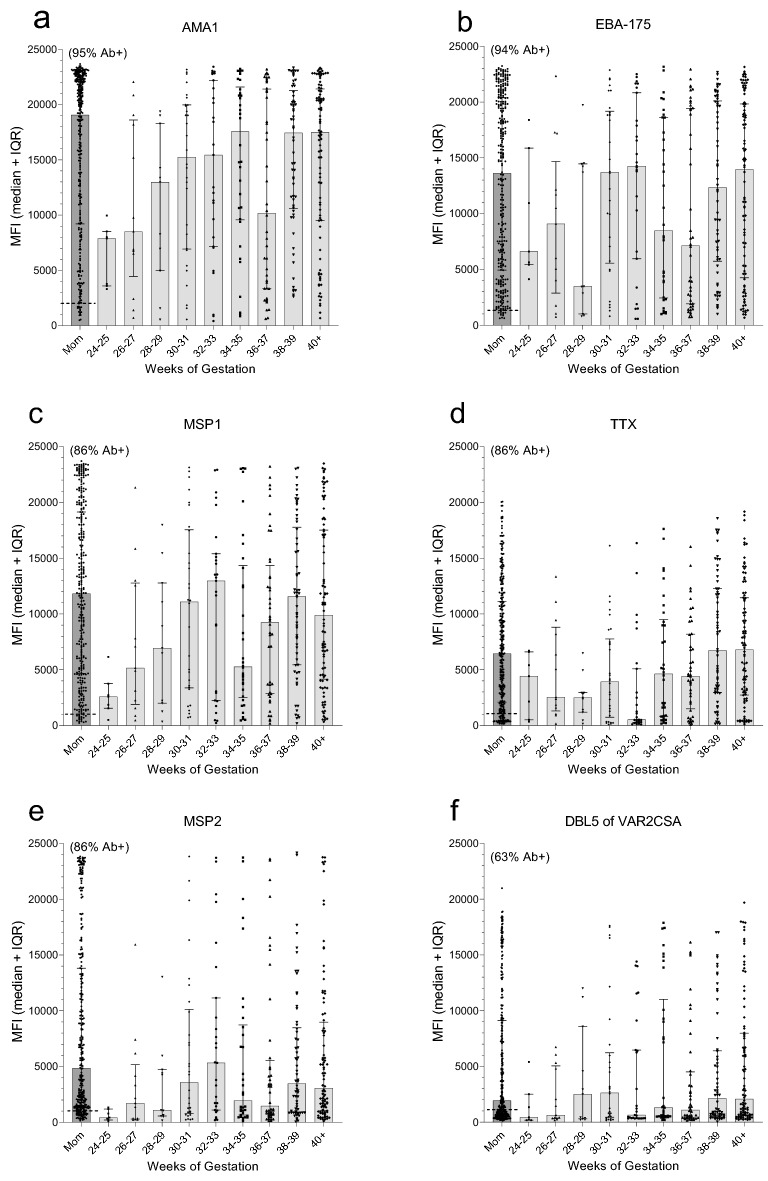

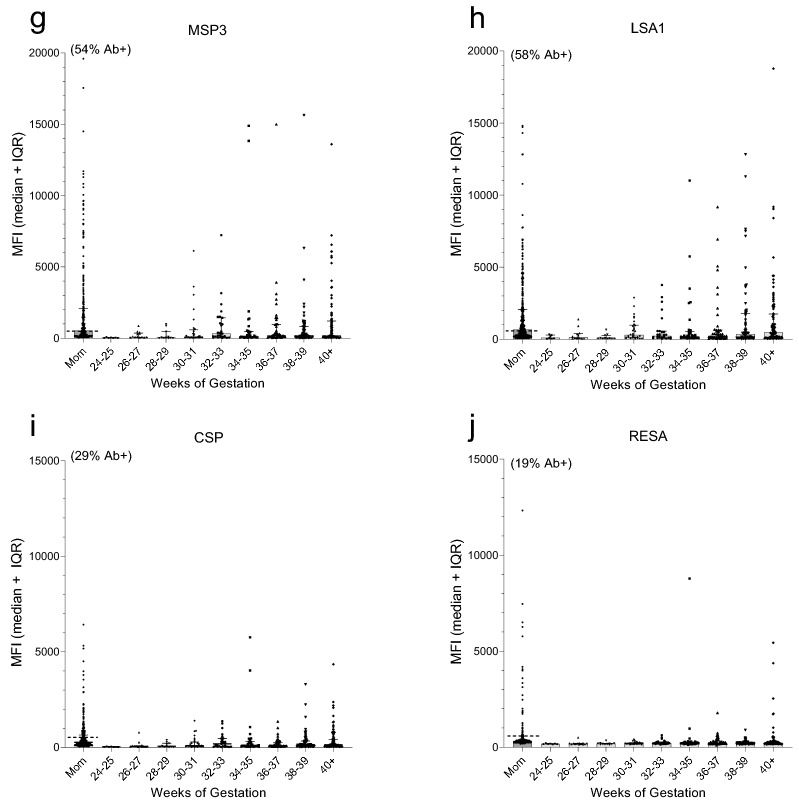
Transfer maternal of antigen-specific IgG during gestation. The first column in panels (**a**–**j**) (Labeled mom) shows (i) the distribution of Abs for the 320 women, (ii) the dotted horizontal line indicates the cut-off for Ab positivity, and (iii) the percentage (%) shown above the column is the percentage of Ab+ mothers. The other 9 columns illustrate the distribution of MFI in cord blood of premature and term babies at two-week intervals based on all 320 babies. Horizontal lines and error bars are the median and IQR. AMA-1: apical merozoite antigen-1, EBA-175: erythrocyte binding antigen-175; MSP1-42: c-terminal 42 kDa fragment of merozoite surface protein-1; TTx: tetanus toxoid; MSP2: merozoite surface protein 2; DBL5: Duffy-binding like domain 5 of VAR2CSA; MSP3: merozoite surface antigen-3; LSA1 liver-stage antigen 1; CSP: circumsporozoite protein; RESA: ring-stage erythrocyte antigen. MFI: median fluorescence intensities.

**Figure 3:**
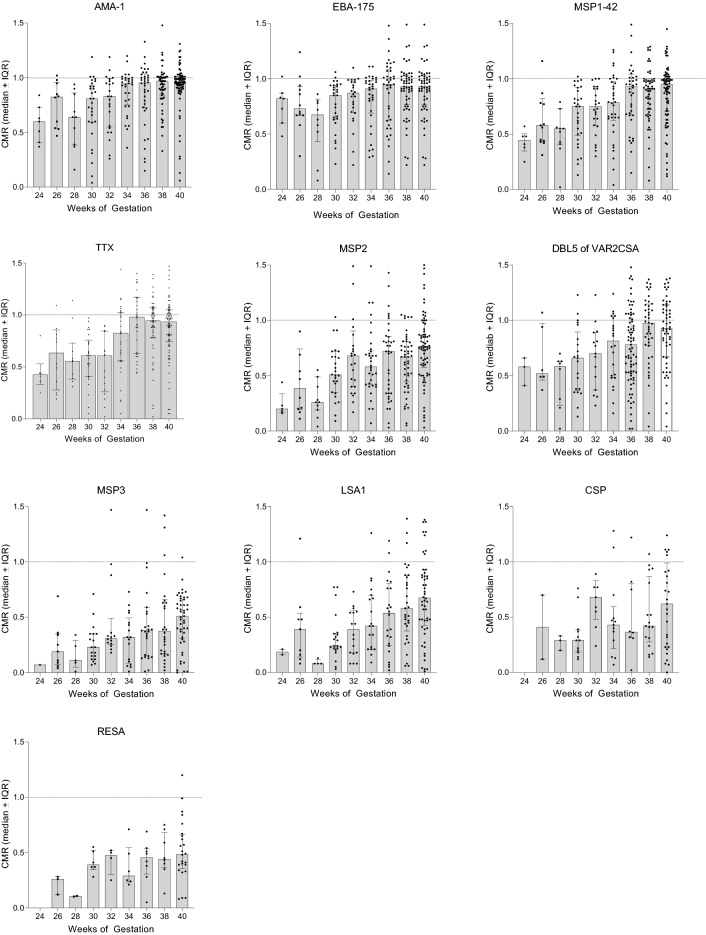
Change in cord-maternal ratio (CMR) with gestational age. Results are based on all Ab-positive mothers for each antigen. Thus, the sample size differs among the antigens. Results are expressed as median ± IQR. Horizontal dotted line highlights CMR = 1.0, i.e., the point where median Ab levels are equivalent in maternal and cord blood.

Next, all 320 paired plasma samples were screened for Abs to 9 malarial antigens and TTx using a multiplex assay, with Ab levels being reported in median fluorescence intensities (MFI) (Fig. 2). Among the mothers, (i) a wide range of Ab levels was detected, (ii) MFI were not normally distributed, (iii) many mothers lacked Abs to some of the antigens, and (iv) Ab levels did not change significantly between 24 weeks of pregnancy and term (i.e., no significant difference across the 9 gestational age groups, (K–W Test: *p* values ranged from 0.18 to 0.76 for the 10 antigens). Accordingly, results for all 320 mothers are combined in the first column of each panel of Fig. 2 (Fig. 2a–j). Overall, > 85% of the mothers had Abs to 4 malarial antigens, i.e., apical merozoite antigen-1 (AMA-1), erythrocyte binding antigen-175 (EBA-175), the c-terminal 42 kDa fragment of merozoite surface protein-1 (MSP1-42), and merozoite surface protein 2 (MSP2), as well as TTx; whereas, > 50% had Abs to the Duffy-binding-like domain 5 (DBL5) of VAR2CSA, the merozoite surface antigen-3 (MSP3), and the liver-stage antigen 1 (LSA1); but < 30% of the women had Abs to B-cell epitopes in the synthetic peptides of the circumsporozoite protein (CSP) and ring-stage erythrocyte antigen (RESA) (Fig. 2a–j). Since only Ab-positive (Ab+) women can transfer Abs transplacentally, some babies did not have Abs to some of the antigens. With increasing gestational age, a clear increase in maternal IgG was observed in cord blood to AMA-1, EBA-175, MSP1-42, TTx, DBL5 and MSP2 (Fig. 2a–e); however, only a slow, minor increase in Ab levels in cord blood was observed to MSP3, LSA-1, CSP, and RESA (Fig. 3g–j). The different patterns of increase may reflect differences in immunogenicity among the antigens.

The proportion of maternal IgG the fetus has received from the mother is shown in Fig. 3. Since only Ab+-mothers can transfer Abs to the fetus, results in Fig. 3 are from Ab+ mothers only. Maternal Ab levels were reached ~ 34–35 weeks for AMA-1, EBA-175, MSP1-42 and TTx; around ≥ 40 weeks for MSP3 and DBL5; but median maternal levels were never reached for MSP3, LSA1, CSP and RESA. Thus, for the latter antigens, newborns had lower Ab levels than their mothers, even though transplacental transfer is an active receptor-mediated process.

#### Comparison of timing of transfer among the antigens

**Figure 4:**
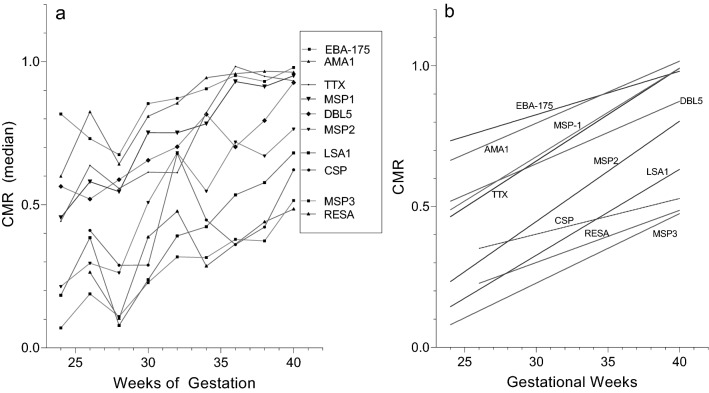
Change in CMR with gestational age. (**a**) For each antigen, median CMR for each gestational age group was plotted. The legend on the right lists the antigens in the order the lines crossed the Y-axis at 40-week. All lines had a significant positive slope (r values: 0.68–0.96), except for CSP (r = 0.43). (**b**) Linear regression of results shown in (**a**) for each antigen from 24 weeks to term. No significant differences among the slopes were detected for the 10 antigens (K–W test: *p* = 0.101); but a significant difference in elevation (intercepts) was evident (*p* < 0.001). Although the slope for the CSP line appears different, the difference was not significant and most likely looks different due to the small sample size (n = 61).

Since differences in the amount of Abs to different antigens transferred to the baby were found, (i) the amount of Ab in the second trimester (24–27 weeks), (ii) the rate of Ab transfer between 24 weeks and term, and (iii) Ab levels at term were determined (Fig. 4, Table 2). In addition, CMR values were plotted against gestational age, curve fitting models were applied, and results showed that linear regression provided the best fit (Fig. 4a,b). Comparisons of the rates of increase of Ab medians (slopes) with gestational age found no differences in the slopes among the 9 malarial antigens and TTx (K–W: *p* = 0.747). That is, the rates of transfer of Abs to the individual antigens did not differ significantly (Table 2-middle column; Fig. 4b); whereas, the elevation of the lines (amount) differed (e.g., range at term: CMR 0.48–0.98) (Table 2 columns 1 & 3, Fig. 4a,b). Overall, Ab amounts and CMR during the second trimester were elevated for AMA1, EBA-175, MSP1, TTx compared to LSA1, CSP and RESA, and remained higher thereafter. Thus, the amount of antigen-specific IgG and CMR at term were established by the second trimester and transfer took place at the same rate thereafter.

**Table 2:** Comparison CMR at the end of second trimester, rate of transfer, and CMR at term.

	Median CMR at 24–27 weeks* (# of samples)	Rate of Transfer** (Increase in CMR/week)	CMR at 40 weeks* (# of samples)
**Antigens**			
AMA-1	0.63 (0.5,0.84) (17)	0.022 ± 0.005	0.96 (0.91,1.0) (87)
EBA-175	0.76 (0.76,0.90) (19)	0.015 ± 0.004	0.93 (0.55,1.1) (71)
MSP-1	0.48 (0.42,0.77) (19)	0.031 ± 0.003	0.95 (0.7,1.0) (83)
TTx	0.47 (0.33,0.80) (18)	0.033 ± 0.005	0.94 (0.74,1.1) (82)
MSP-2	0.22 (0.18,0.47) (15)	0.036 ± 0.006	0.76 (0.55,0.95) (73)
DBL5-VAR2	0.55 (0.45,0.80) (9)	0.022 ± 0.004	0.93 (0.67,1.1) (55)
MSP-3	0.15 (0.07,0.35) (10)	0.025 ± 0.003	0.51 (0.31,0.66) (49)
LSA-1	0.21 (0.16,0.48) (11)	0.031 ± 0.007	0.66 (0.43,0.89) (52)
CSP	(0)	0.013 ± 0.011	0.62 (0.23,0.99) (29)
RESA	0.26 (0.12,0.28) (3)	0.019 ± 0.008	0.48 (0.35,0.67) (24)

*Median (IQR) for 24–27 weeks (i.e., end of the second trimester) and CMR at ≥ 40 weeks (i.e., term) are provided. Numbers in (#) indicate sample size.

**The increase in CMR per week of gestation ± standard error, between 24 and 40+ weeks, is shown. The numbers in parentheses (#) indicate sample size.

### Influence of HIgG and PM on transfer of total IgG

**Figure 5:**
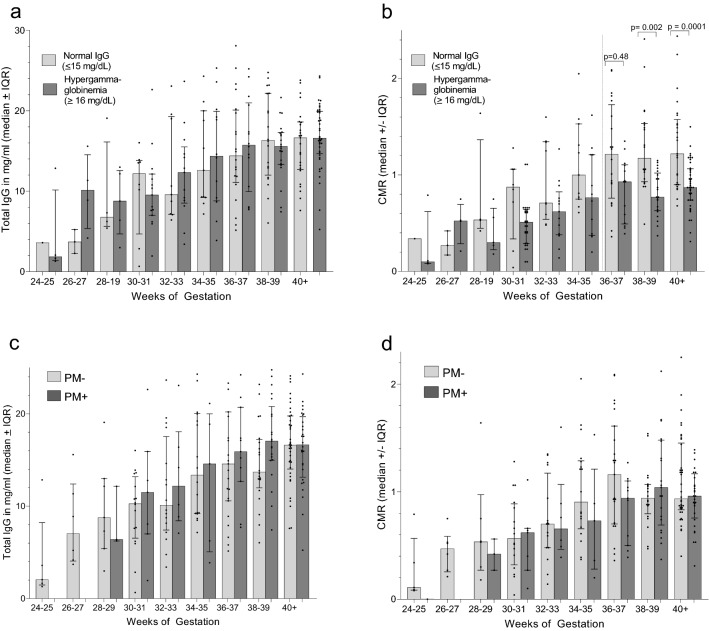
Influence of hypergammaglobulinemia (HIgG) and placental malaria (PM) on transplacental transfer of total IgG. (**a**) and (**b**) Comparison of total IgG levels and CMR in cord blood between babies born to mothers with normal IgG levels (≤ 15 mg/ml IgG) and hypergammaglobulinemia (≥ 16 mg/ml). (**c**) and (**d**) Comparison of the effect of PM malaria on total IgG and CMR. Results are expressed as median ± IQR. *P* values for all comparisons with significant differences (*p* ≤ 0.05; using the Mann–Whitney test for pair-wise comparisons) are provided.

Maternal and cord pairs were stratified into two groups based on maternal IgG levels; i.e., mothers with IgG ≤ 15 mg/ml (n = 90) and mothers with HIgG ≥ 16 mg/ml (n = 140) (Fig. 5a,b). No significant difference in pairwise comparisons of IgG levels in cord blood with gestational age was found between infants whose mothers had normal and HIgG (*p* values ranged from 0.11 to 0.90 for different age groups) (Fig. 5a); however, CMR were generally lower when mothers had HIgG, with differences becoming significant at 36–37 weeks until term (Fig. 5b). Thus, the amount of IgG in cord blood was similar throughout gestation in infants whose mothers had normal and HIgG; whereas, the calculated CMR were reduced in the HIgG groups due to higher denominators. These results demonstrate that IgG transfer per se was not altered due to HIgG and that low CMR do not indicate a defect in the FcRn transport system. Rather, low CMR are due to the way CMR are calculated.

The influence of PM was also evaluated in PM^+^ (n = 81) and PM^−^ (n = 149) women (Fig. 5c,d). Although the amount of IgG was significantly elevated in PM+ compared to PM− mothers at delivery (PM+ 18.1 ± 0.45 mg/ml; PM− 15.8 ± 0.33 mg/ml, p < 0.001), IgG levels in the newborns did not differ significantly between PM^+^ and PM^−^ newborns, even at term (Fig. 5c, all paired *p* values > 0.5). Even with the increase in maternal IgG in PM+ mothers, CMR did not differ significantly among the newborns (all paired *p* values > 0.12) (Fig. 5d). Thus, the increase in IgG in PM+ women was inadequate to significantly impact the CMR. Since placental pathology is often more severe in primigravidae, total IgG results were stratified into 4 groups, i.e., (i) primigravidae-PM−, (ii) primigravidae-PM+, (iii) multigravidae (MG) PM−, and (iv) MG MP+. Across group comparison revealed no difference in the distribution of CMR (Kruskal–Wallis test: *p* = 0.907) with median CMR of 0.99, 1.03, 1.00 and 1.04, respectively. Overall, there was no evidence that placental malaria reduced the transfer of maternal IgG during pregnancy.

### Influence of HIgG and PM on transfer of antigen-specific Abs

**Figure 6:**
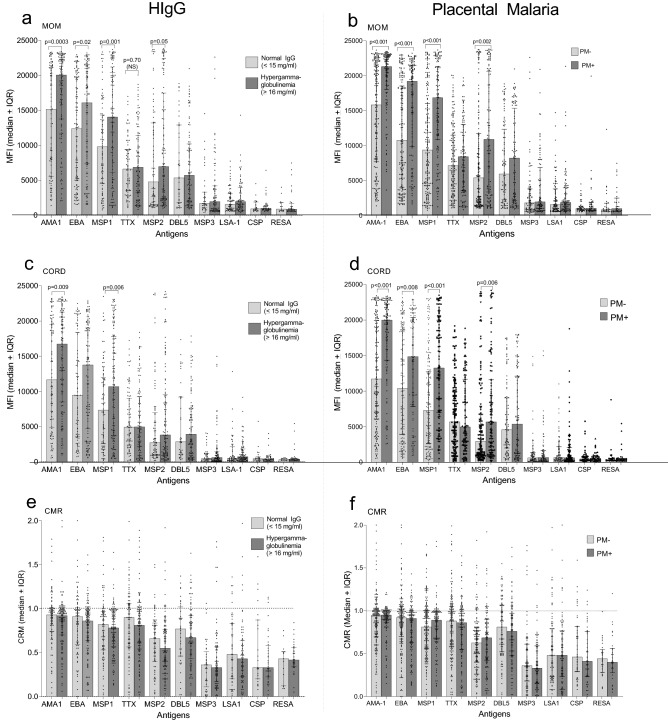
Influence of hypergammaglobulinemia (HIgG) and placental malaria (PM+/PM−) on transfer of maternal IgG Abs to malarial antigens and tetanus toxoid. All *p* values < 0.05 for pair-wise comparisons (Mann–Whitney test) are shown. Dotted horizontal lines are reference markers for when mother and baby IgG levels are equivalent. Results include all Ab-positive mothers for each antigen. (**a**–**d**) are based on 230 pairs; whereas, (**e**–**f**) are based on samples from Ab-positive mothers.

Compared to PM^−^ women and those with normal IgG levels, Ab levels were significantly higher in pregnant women with HIgG and PM^+^ for AMA1, EBA-175, MSP-1, and MSP2 (all *p* values < 0.05), but not DBL5, MSP3, LSA1, CSP and RESA (Fig. 6a and b). As expected, neither condition had an impact on Abs to TTx. Likewise, increased amounts of Abs to AMA1, EBA-175, MSP-1, and MSP2 were also detected in babies of mothers with HIgG and PM+ (all *p* values < 0.05), but not the other antigens (Fig. 6c,d). Thus, HIgG and PM^+^ resulted in increased, not decreased, amount of Abs the baby had to some antigens (namely, those with MFIs > 5000), but not other antigens. Neither HIgG nor PM^+^ altered CMR (Fig. 6e,f) and were in the same range as those shown in Table 2, i.e., antigens with MFI > 5000, median CMR ranged from ~ 0.7 to 0.95 and were lower for antigens with MFI < 5000 (range 0.35–0.45) (Fig. 6e,f). Thus, newborns of mothers with HIgG and PM+ were more likely to have higher amounts of Ab to major antigens (i.e., AMA1, EBA-175, MSP-1, MSP2) than babies of mothers with normal IgG or who were PM−, but similar amounts of the other antigens.

## Discussion

Although the precise role of maternal Abs in protecting newborns from malaria is unclear, Abs are the major mechanism of protection against severe blood-stage *P. falciparum* infections in children and adults. Accordingly, pediatricians are faced with deciding if a premature newborn would benefit from receiving chemoprophylactic treatment for malaria or other diseases. Results from the current study suggest that premature Cameroonian babies, who are born after 34–36 weeks or weighing over 2000–2500 g, will have total IgG levels similar to their mothers (Fig. 1). However, very premature babies born < 34th weeks, have reduced Abs.

Transplacental transfer of maternal IgG starts as early as 6 weeks, then slowly increases with fetal levels reaching 5–10% of maternal IgG levels by 17–22 gestational weeks^10^. As expected, very premature Cameroonian babies at 24–25 weeks had ~ 15% of maternal IgG levels (Fig. 1A). The precise timing of IgG transfer between the 24th and 34th week has been difficult to study. Using cord blood samples collected in the USA and Europe from normal pregnancies, both curvilinear and linear increases have been reported, with maternal levels reached by ~ 33–36 weeks^2,22–24^. Results in Fig. 4, based on data from premature Cameroonian babies, show a linear increase between 24 weeks and term (Fig. 4, Table 2), with maternal levels reached ~ 34–35 weeks of gestation. Thus, results from Cameroon are in agreement with those reported elsewhere^22–24^.

Antibody concentration has an impact on transplacental transfer^8,25–27^. Calculations from 6 studies in westernized countries, at term mothers averaged (± SEM) 10.5 ± 1.8 ml/ml IgG, cord blood had 12.8 ± 1.3 mg/ml, resulting in CMR ratios of 1.2 ± 0.2^14,22,23,28–30^ In comparison, calculations based on data from 9 African studies for mothers averaged 17.3 ± 3.1 mg/IgG, cord blood 14.7 ± 2.5 mg/ml, with CMR of 0.85 ± 0.1^5,13,14,16,30–34^. Thus, African newborns have higher IgG levels that babies in westernized countries, but CMR are lower due to elevated maternal IgG. Several recent review articles have reported that maximal levels of IgG are reached in cord blood around ~ 15 mg/ml, suggesting that the FcRn has become saturated^25,26^. Results in Fig. 1 show similar results, namely that once cord blood IgG concentrations reach ~ 15–16 mg/ml at 34–35 weeks, IgG levels no longer increase, indicating that the physiological carrying-capacity of FcRn has been reached. An upper limit of baby IgG is expected since IgG molecules not bound to FcRn are degraded during transcytosis. Accordingly, CMR would most likely be < 1.0 when mothers have IgG levels > 15 mg/ml, i.e., hypergammaglobulinemia.

Because the FcRn randomly binds the Fc-region of IgG, one would predict that antigenic-specificity would not influence efficacy of transplacental transport and CMR would be similar for all antigens. However, such is not the case. For example, both HIgG (concentration) and PM (infection) have been associated with reduced transfer of IgG to some pathogens (e.g., herpes simplex, respiratory syncytial, varicella-zoster, and measles), but not others (diphtheria toxoid, *S. pneumonia*, *Hemophilis influenza* type b, and TTx)^21^. In the current study, CMR at term ranged from 0.48 to 0.98 for 9 malarial antigens within the same pathogen (Table 2). Similar results have been reported for other *P. falciparum* antigens^16,35^ and reviewed in^17^. Transplacental transport can be influenced by (i) Ab concentration, (ii) isotype, (iii) placental integrity, and (iv) infectious diseases. IgG1 preferentially crosses the placenta, followed by IgG3, IgG4 = IgG2^7,25^. With the exception of MSP3 (IgG3 biased), the other 8 malarial Abs and TTx were predominantly IgG1. Thus, isotype does not seem to explain the difference, except for MSP3. Based on random selection, Abs in high concentration would have a greater probability of being captured by the FcRn than those in low concentration. However, numerous studies have reported that the efficacy of transport (CMR) is often lower when mothers have high Ab titers for specific antigens. In fact, a negative linear relationship was reported between maternal Ab titers to many viral and bacterial antigens and CMR^8,25,30^. Thus, sampling bias does not explain the large variation in CMR. Although the difference of efficacy of transport is well recognized, the reason for the difference is not clear, but suggests that some type of antigenic-specificity may take place.

To our knowledge, this is the first study to show that differences in CMR can be detected as early as 24–25 weeks of gestation (Table 2-column 1), with CMR ranging from 0.22 to 0.76 for the 9 malarial antigens and TTx. Since the rate of transport of Abs to the antigens was the same during gestation (Fig. 4), events prior to the 24th week must preferentially allow Abs to some antigens to cross the placental barrier. As a consequence, the relative proportion of antigen-specific Abs at term was established early in gestation. Events influencing unequal transport of antigen-specific Abs are unclear, but between the 13th and 24th weeks a number of developmental changes take place in the placenta. For example, the cytotrophoblast barrier begins to breakdown and FcRn on syncytiotrophoblasts become exposed to maternal blood allowing active transport to begin. During the second trimester, maternal cells and DNA traffic to the fetus either passively by microtransfusion or actively transferred via the VEGF-A gradient across the placenta^36,37^. Likewise, at 20 weeks malarial antigens can cross the placenta and stimulate an IgM Ab response in the developing fetus^38^. How malarial antigens reach the fetal blood is unclear, but immune complexes do not appear to be involved^38^. Thus, early in the second trimester it is possible that some form of facilitated diffusion occurs that allows Abs, most likely those in the highest concentrations, to cross the syncytiotrophoblasts layer, diffuse through the stroma, and be transferred into fetal vessels. Clearly, more information about how substances are moved across the placenta between the 13th and 28th weeks is needed.

Depending on the immune status of the mother, PM may cause a variety of changes in the intervillous space, from little or no pathology in immune mothers to extensive inflammation including infiltration of mononuclear cells, increased levels of proinflammatory cytokines, and altered VEGF levels, especially in primigravidae. It is therefore not surprising that various results have been reported for the influence of PM on transport of IgG to malaria and other pathogens. Harrington et al. summarized the results from 17 studies of the effect of PM on Ab transfer for 13 malarial, 8 viral and 3 bacterial antigens and documented the diversity of results that have been reported^17^. In general, PM (i) can increase, decrease or have no effect on absolute Ab levels to malarial antigens in cord blood, (ii) reduce or have no effect on CMR to malaria-specific antigens, (iii) have no effect or reduced absolute Ab levels to viral and bacterial antigens, and (iv) reduce or have no effect on CMR to viral antigens. Similar results have been reported in other studies not included in the review. One interesting study conducted outside of Africa, reported decreased Ab levels in cord blood of primigravidae (where pathology is most likely to be the greatest), but not multigravidae, to AMA1, EBA-175, MSP2 and DBL5^39^. The timing of *P. falciparum* infection during pregnancy is also important, since boosting of Ab toward the end of pregnancy will boost antimalarial Ab titers, thereby increasing IgG transfer. In the current study, median Ab MFI to the malarial antigens were either higher or same in babies of PM+ compared to PM− mothers (Fig. 6b). The finding that PM does not alter IgG transport in this study was not surprising since 72% of PM+ women were multigravidae and likely to have little placental pathology. To date, the only suggested pathological change in the placenta associated with reduced CMR was infiltration of hemozoin-laden macrophages in the placentas of primigravidae in Papua New Guinea^39^. Because of the large number of variables, it is not surprising that different studies have reached different conclusions about the influence of PM on transplacental transport of maternal IgG.

In summary, to our knowledge this is the first study to explore transplacental transfer of IgG in premature African newborns. Data suggest that babies born < 34–35 weeks are likely to have reduced amounts of maternal Abs to malaria and other pathogens and that the relative amounts (CMR) differed among the antigens. These results have important implications for scientists developing maternal immunization programs in Africa.

## Methods

### Study population

Samples were collected at delivery between 1996 and 2001 in Yaoundé, Cameroon at the Biyem Assi District Hospital and Central Maternity Hospital^40^. At this time, *P. falciparum* transmission was perennial in the city with individuals receiving an estimated 13 infectious mosquito bite per person per year. Approximately, 27.8% of primigravidae and 15.6% of multigravidae had PM at delivery^40^. The study was completed before intermittent preventive treatment (IPTp) was introduced into Cameroon. Information on the mother’s age, gravidity, and the baby’s birth and placental weights were recorded. Gestational length was assigned based on last menstrual period, sonogram results when available, and assessment by the obstetrician. Informed consent was obtained from the mothers at delivery and the study was carried out in accordance with relevant guidelines and regulations. Archival plasma samples and information in a password-protected database were available for use in the current study. All available paired maternal and cord plasma samples (n = 166) from PTD (< 38 week) were selected and divided into 7 groups based on 2-week intervals of gestational age. In addition, 154 pairs were randomly selected from women with term deliveries who were PM-positive (n = 77) and PM-negative (n = 77), to ensures an adequate sample size for testing the influence of PM on IgG transfer. Characteristics of the 320 women are shown in Table 1.

### Ethical approval for human subjects

Ethical approval for recruitment, enrollment, and sample collection was obtained from the National Ethics Committee, Cameroon and the Institutional Review Board of Georgetown University (1994-158). All participants provided written informed consent and all aspects for the study were carried out in accordance with relevant guidelines and regulations. Use of archival deidentified samples in the current study was approved by the Committee on Human Subjects, University of Hawai’i (CHS#18199).

### Cord and maternal blood samples

The placenta was placed maternal-side down, the umbilical cord was clamped at the basal end, and fetal cord blood was drawn in the presence of heparin. The placenta was subsequently re-positioned maternal-side up and a tissue biopsy (2 cm × 2 cm × 2 cm) was excised midway between the site of cord insertion and the edge of the placental disc. Maternal venous blood was collected into heparinized tubes.

### Detection of placental malaria

Thick and thin blood smears of cord and maternal blood were prepared, and impression smears of the placental biopsy were made. Slides were stained with Diff-Quik (Baxter Scientific Products) and examined for the presence of *P. falciparum*-infected erythrocytes (IE). Two thousand erythrocytes were examined before a slide was determined to be negative. When positive, parasitemia was recorded as percent of infected erythrocytes determined from impression smears.

### Antigens-coupled microspheres for detection of IgG Abs

Malaria antigens included the following recombinant proteins: apical merozoite antigen-1 (AMA-1 3D7 strain) expressed in yeast (mw: 83,000) and merozoite surface protein-1 C-terminal region (MSP-1_42_ 3D7 strain) expressed in *Escherichia coli* (mw: 42,000) provided by the Malaria Vaccine Development Branch, NIAID, NIH; the erythrocyte-binding antigen-175 region II (EBA-175) expressed in yeast (mw: 60,000) from Science Applications International Corp., Frederick, MD; merozoite surface protein-2 (MSP-2 3D7 strain) without the N-terminal signal and C-terminal GPI-attachment sequence, but containing an N-terminal six-HIS tag from R. Anders, La Trobe University; merozoite surface protein-3 (MSP-3) C-terminal region expressed in *E. coli* (mw: 22,000) from P. Druihle, The Pasteur Institute; and the Duffy Binding-Like 5 (DBL5x) domain of VAR2CSA expressed in baculovirus (mw: 45,000) provided by A. Salanti, University of Copenhagen. The following peptides were custom synthesized by AnaSpec, Inc.: the circumsporozoite protein (CSP) consisting of 5- PNAN repeats (mw: 2,100) with an N-terminal cystine coupled to bovine serum albumin (BSA); ring-stage erythrocyte surface antigen (RESA) consisting of 5 EENV repeats (mw: 2500) coupled to BSA through an added cystine residue; and a 40-mer peptide from liver-stage antigen-1 (LSA-1) (mw: 4500) AKEKLQGQQSDLEQERLAKEKLQEQQSDLEQERLAKEKL. Tetanus Toxoid USP (Adventus Pasteur Inc.) was used.

A broad-array of malarial antigens was selected from different parasite stages including merozoites (AMA-1, EBA-175, MSP1-42, MSP2, MSP3), the ring-stage (RESA), trophozoites (DBL5-VAR2CSA), sporozoites (CSP), and the liver-stage (LSA1). The antigens also differ in immunogenicity, for example, AMA1, EBA-175, MSP1-42 induce high titers in malaria endemic areas; whereas, only one or two repetitive and/or immunodominant epitopes have been identified in other antigens (EENV in RESA, NANP in CSP and a 39-mer sequence in LSA1). Immune protection is not mediated by a single antigen, but rather Abs to a combination of antigens, including those used in this study, working together to reduce severity of disease and decrease the number of pre-erythrocytic stage parasites. By using a diverse panel of antigens from different parasite stages with different immunogenicity it was envisioned that a more complete picture of transplacental IgG transfer could be obtained.

### Coupling antigens to the microspheres

Antigens were coupled to microspheres using protocol provided by the manufacturer, as described previously by Fouda et al.^41^. In brief, SeroMAP Microspheres (Luminex Corp.) with different spectral addresses were activated and incubated with 1 µg of EBA-175, MSP-2 and MSP-3; 5 µg of AMA-1, MSP-1 and DBL5; 12.5 µg of CSP-BSA and RESA-BSA, 112.5 µg of LSA-1, and 50 µl of TTx overnight. The coupled microspheres were washed and stored in phosphate buffered saline containing 0.05% sodium azide until used.

### Multiplex assay for measuring Ab levels

The multiplex assay was performed as previously described^41^. Briefly, 50 μl of maternal or cord plasma (diluted 1:500) was mixed with 50 µl pooled microspheres (3000 microspheres/antigen), incubated 1 h, washed, and then incubated with 100 µl of 2 µg/ml of R-phycoerythrin-conjugated, F(ab')_2_ fragment, goat anti-human IgG (Fcγ fragment specific) (Jackson ImmunoResearch). The microspheres were washed and read in a Liquichip M100 reader (QIAGEN). Results were expressed as median fluorescence intensity (MFI). The positive control consisted of 36 plasma samples pooled from multigravidae used at 3 dilutions. Negative controls was pooled plasma from 90 individuals in the USA not exposed to malaria.

### Determining total IgG levels

The total amount of IgG was determined for the first 230 paired mother-cord samples (n = 123 PTD and n = 107 FTD) using an Ab capture assay. First, 1 µl of goat anti-human IgG was coupled to 5 million microspheres as described above. Then, 50 µl of plasma (diluted 1:150,000) was combined with 50 µl anti-human IgG-coated microspheres (3000 microspheres/50 μl) and incubated for 1 h at 500 rpm, washed 3-times with PBS. Finally, microspheres were incubated with 100 µl of the secondary anti-human IgG Ab described above for 1 h, washed, and read in LiquiChip M100 reader. An 8-point standard curve (mg/ml) was developed using plasma from Cameroonian subjects with known levels of IgG determined by nephelometry.

### Statistical analysis

Demographic, clinical and parasitological characteristics were summarized by descriptive statistics: means with standard errors (SEM) for continuous variables, i.e., maternal age, and percentages for categorical variables, i.e., primigravidae and malaria status. To compare differences among the gestational age groups with continuous variables (i.e., mg/ml IgG and MFI that were not normally distributed) the non-parametric Kruskal–Wallis (K–W) test was used. When overall significance was identified, pair-wise comparisons using the Mann–Whitney test was applied. Differences between proportions (e.g., percent primigravidae) were compared using Chi Square. Samples were considered to be Ab-positive when MFI were greater than the mean + 3 Standard Deviation (SD) of the negative control. Ab levels were considered to be high if MFI were between 5000 and 20,000 and low if MFI were above cut-off but < 5000 MFI. To determine the rate of Ab transfer (slope), median CMR were plotted against gestational age, standard linear regression was used, and slopes and elevations were compared using K–W. Statistical significance was determined by *p* < 0.05.

## Data Availability

The datasets generated during and/or analysed during the current study are available from the corresponding author on reasonable request.
